# Nutritional evaluation and support for children infected with HIV

**DOI:** 10.1590/S1516-31802000000500002

**Published:** 2000-09-01

The nutritional deficiencies from infection by HIV are frequently more serious in children than in adults, due to children's need to demand additional nutrients for their growth and development. The compromising of nutrition is related to the increase in the number of episodes of infectious complaints and also contributes to a slower recovery from these. In the United States in 1994, the so-called “wasting” syndrome, a serious condition of denutrition accompanied by weight loss, diarrhea and/or fever, affected 17% of children infected by HIV.^1^ Around 40% of the 300 children infected by HIV and admitted into the Instituto da Criança in the period from 1985 to 1998 presented a history of weight loss and/or denutrition at diagnosis.

The article “Evolution of nutritional status of infants infected with Human Immunodeficiency Virus”,^2^ presented in this issue, forms a relevant contribution towards the knowledge of nutritional conditions among Brazilian children exposed perinatally to HIV. From the evaluations of the series, the article demonstrates the early compromising of growth among the infected children, comparing the Z-scores (weight/age, height/age and weight/height) with those of non-infected (seronegative) children.

This evidence can be associated to that of another recent publication:^3^ a prospective evaluation of the energy balance and anthropometric analysis among unweaned children of mothers seropositive to HIV, which revealed significant compromising of nutrition in the infected children and average values for energy expenditure at rest that were 30% greater than those observed in the non-infected infants. Both works conclude that in clinical practice early nutritional intervention must be commended so as to guarantee the maintenance of immunological equilibrium and muscle mass, and a better quality of life for the children infected by HIV.

One wide-ranging proposal for nutritional support for such a clientele is presented in two very recent reviews.^4,5^ They emphasize that the attendance must be provided by a multidisciplinary team, with the inclusion of a professional in the field of nutrition. Careful guidelines for the approach must be drawn up that include:

- The analysis of the anthropometric data (weight, height, brain perimeter, arm circumference, measurements of the triceptal and subscapular creases) at the start of the follow-up and then at every 1 to 3 months depending on the age and general conditions of each child, and biochemical evaluation;
- Record-keeping of alimentation and protein-caloric calculation of food ingested, as well as analysis of the adequacy of the menu;
- Offer of a diet with raised levels of protein and calories, and with an appropriate composition of other nutrients and vitamins. The energy needs are expected to reach between 100% and 150% of the Daily Recommended Calculation, remembering that these depend on the age and the intercurrences presented by the patients;
- Nutritional intervention must be started early when faced with any evidence of alterations in the evolutive parameters, and also when an increase in energy demand is anticipated, with the use of nutritional supplements (commercially available formulae). The use of appetite stimulators for short periods, such as megestrol acetate, appears to be useful in some cases. When there is a failure in oral intervention, methods that can be considered range from the use of nasogastric probe to gastrostomy, depending on the patient and his clinical situation.

The survival of HIV-infected children has increased significantly over recent years since the introduction of combined anti-retroviral therapy allied to the improvement in medical care and thus, to promote normal growth, avoiding weight loss must be a priority in the care of the HIV-infected child.
